# Is Hemoglobin Level in Patients with Nasopharyngeal Carcinoma Still a Significant Prognostic Factor in the Era of Intensity-Modulated Radiotherapy Technology?

**DOI:** 10.1371/journal.pone.0136033

**Published:** 2015-08-27

**Authors:** Shan-Shan Guo, Lin-Quan Tang, Qiu-Yan Chen, Lu Zhang, Li-Ting Liu, Pei-Yu Huang, Ka-Jia Cao, Ling Guo, Hao-Yuan Mo, Xiang Guo, Ming-Huang Hong, Mu-Sheng Zeng, Chao-Nan Qian, Hai-Qiang Mai

**Affiliations:** 1 Sun Yat-sen University Cancer Center;State Key Laboratory of Oncology in South China, Collaborative Innovation Center for Cancer Medicine, Guangzhou, Guangdong, P. R. China; 2 Department of Nasopharyngeal Carcinoma, Sun Yat-sen University Cancer Center, Guangzhou, Guangdong, P. R. China; 3 GCP Center, Sun Yat-sen University Cancer Center, Guangzhou, P. R. China; University Campus Bio-Medico, ITALY

## Abstract

**Background:**

Hemoglobin (Hb) levels are regarded as an important determinant of outcome in a number of cancers treated with radiotherapy. However, for patients treated with intensity modulated radiotherapy (IMRT), information regarding the prognostic value of hemoglobin level is scarce.

**Patients and Methods:**

A total of 650 patients with nasopharyngeal carcinoma (NPC), enrolled between May, 2005, and November, 2012, were included in this study. The prognostic significance of hemoglobin level (anemia or no-anemia) at three different time points was investigated, including before treatment, during treatment and at the last week of treatment. Univariate and multivariate analyses were conducted using the log–rank test and the Cox proportional hazards model, respectively.

**Results:**

The 5-year OS (overall survival) rate of patients who were anemia and no-anemia before treatment were 89.1%, and 80.7% (P = 0.01), respectively. The 5-year DMFS (distant metastasis-free survival) rate of patients who were anemia and no-anemia before treatment were 88.9%, and 78.2% (P = 0.01), respectively. The 5-year OS rate of patients who were anemia and no-anemia during treatment were 91.7% and 83.3% (P = 0.004). According to multivariate analysis, the pre-treatment Hb level predicted a decreased DMFS (P = 0.007, HR = 2.555, 95% CI1.294–5.046). Besides, the mid-treatment Hb level predicted a decreased OS (P = 0.013, HR = 2.333, 95% CI1.199–4.541).

**Conclusions:**

Hemoglobin level is a useful prognostic factor in NPC patients receiving IMRT. It is important to control the level of hemoglobin both before and during chemoradiotherapy.

## Introduction

Nasopharyngeal carcinoma (NPC) is rare globally, but it is epidemic in southern China and Southeast Asia [1]. The crude incidence of NPC was 3.61 cases per 100,000 people in 2009 according to data from regions covered by cancer registries [2]. The vast majority of NPCs are poorly differentiated, while a minority are well-differentiated squamous-cell carcinomas [3]. There is a strong association between Epstein-Barr virus (EBV) infection and NPC [3–5]. Radiotherapy is the primary therapy used and, when applied in combination with chemotherapy, is regarded to be the first-line treatment for locoregionally advanced NPC [6, 7].

Hemoglobin (Hb) levels are regarded as an important determinant of outcome in a number of cancers treated with radiotherapy, particularly gynecological tumors and head and neck cancers [8–10]. Several studies have shown a positive relationship between Hb level and survival outcomes after three-dimensional radiotherapy in NPC [11–13]. However, patients received conventional radiotherapy with or without concurrent chemotherapy in upon studies.

The increasingly widespread use of IMRT technology in NPC patients over the past decades has improved the treatment outcomes when compared with conventional radiotherapy, especially in local disease control [14–16]. Information regarding the prognostic value of hemoglobin levels for patients treated with intensity modulated radiotherapy (IMRT) is, however, scarce. Therefore, it is important to explore the prognostic value of Hb in NPC patients in the setting of IMRT. We conducted a retrospective study in NPC patients treated with IMRT, to investigate the significance of hemoglobin level on the outcome of improved radiotherapy treatment.

## Methods and Materials

### Patients and variables

A total of 650 patients (of which 473 were male and 177 were female, with a sex ratio of 2.7:1) who met the following criteria for NPC between May 2005 and November 2012 were included in this retrospective study: (1) histologically proven NPC by biopsy of the nasopharynx; (2) no distant metastasis; (3) no treatment prior to admission; (4) no other tumours or serious illnesses; (5) Eastern Cooperative Oncology Group (ECOG) performance score ≤2; and (6) received radical IMRT during the course of treatment. Participants underwent a pre-treatment evaluation that included a complete patient history, a routine physical examination, computed tomography (CT) or magnetic resonance imaging (MRI) of the head and neck, a fibre optic endoscope examination of the nasopharynx, and haematological and biochemical tests. Chest radiography, abdominal ultrasonography and bone scintigraphy were used to exclude distant metastases. All participants were restaged according to the 2010 Union for International Cancer Control (UICC) staging system. The present study was approved by the Institutional Review Board of our institution, and written informed consent was obtained from each patient.

The Hb value of patients was detected in each week since treatment. Pre-treatment Hb level was defined as the Hb value detected before treatment. The mid-treatment Hb level was calculated as the average of Hb levels measured in the first week before radiotherapy through the last week of treatment. The post-treatment Hb level was defined as the Hb value at the last week of treatment. Anemia was defined according to World Health Organization criteria as hemoglobin <130 g/L in men and <120 g/L in women. No patients received erythropoietin therapy. Individual difference value (ΔHb) of Hb level from pre-treatment to post-treatment was equal to (pre-treatment Hb value)-(post-treatment Hb value). Hb continuous decrease was defined as pre-treatment Hb > mid- treatment Hb > post- treatment Hb.

### Treatment

Patients were treated with radical IMRT delivered as five fractions per week. Target volumes were contoured according to our institutional treatment protocol [17], in agreement with the International Commission on Radiation Units and Measurements Reports 50 and 62. The patients received a total radiation dose of 68–70 Gy to the gross tumor volume given in 30–33 fractions (2.12–2.33 Gy per fraction). The radiation dose to the neck lymph node was 66–70 Gy. The overall treatment time was 6–7 weeks. The remaining 646 patients of stage II-IV received 2–3 cycles of concurrent chemotherapy with cisplatin (80–100 mg/m^2^) delivered every 21 days. Four patients at stage I received radiotherapy alone.

### Follow-up

After the completion of treatment, patients were followed up every 3 months throughout the first 3 years, every 6 months for the next 2 years and annually thereafter. The follow-up duration was calculated from the first day of treatment to either the day of death or the day of the last examination. The median follow-up period was 49 months (range, 1.3–85.5 months).

### The endpoints of this study

Overall survival (OS) was defined as the time from the date of treatment to death from any cause or until the date of the last follow-up. Localregional recurrence-free survival (LRFS) was defined as the time from the date of treatment to the absence of a primary site or neck lymph-node relapse or until the date of the last follow-up. Distant metastasis-free survival (DMFS) was defined as the time from the date of treatment to the date of the first observation of distant metastasis or until the date of the last follow-up.

### Statistics

Survival curves were estimated using the product limit method of Kaplan–Meier with the log-rank test. Univariate analysis was conducted using the log-rank test, while multivariate analysis used the Cox proportional hazards model. Age (age ≤ 45 years, >45 years), gender, T stage, N stage, clinical stage, Epstein-Barr Virus (EBV) DNA (<4000 copies/ml, ≥4000 copies/ml), Hb continuous decrease (continuous decrease, no-continuous decrease), ΔHb (<2.2 g/dl, ≥2.2 g/dl), pre-treatment Hb level (anemia, no-anemia), mid-treatment Hb level (anemia, no-anemia), and post-treatment Hb level (anemia, no-anemia) were regarded as factors in the Cox proportional hazards model. Statistical analyses in this study were conducted using SPSS (version 17.0, Chicago, IL, USA). All *P* values were 2-sided. *P*≤0.05 was considered statistically significant.

## Results

### Patient characteristics

The population of this study consisted of a sequential series of 650 patients treated with IMRT from May, 2005 to November, 2012, for NPC. The characteristics of patients in the present study are described in Table 1. Of the 650 patients, 473 were male and 177 were female, with a sex ratio of 2.7:1. The median age was 45 years, ranging from 13 to 79. 620 (95.4%) patients had WHO classification type III undifferentiated carcinomas, and 30 (4.6%) had type II. All the 650 patients had an ECOG score of 1. According to the 2010 UICC staging system of NPC, 4 (0.6%) patients were at stage I, 59 (9.1%) were at stage II, 429 (66.0%) were at stage III and 158 (24.3%) were at stage IV A-B.

**Table 1:** Baseline characteristics of 650 patients with locoregionally advanced nasopharyngeal carcinoma. *Abbreviations*: WHO = World Health Organization; yr = year; EBV = Epstein-Barr Virus.

	No-anemia	Anemia	
	585(%)	64(%)	*P* value
Age(yr),			0.479
<45	315(53.8)	32(49.2)	
≥45	270(46.2)	33(50.8)	
Gender			0.006
Female	150(25.6)	27(41.5)	
Male	435(74.4)	38(58.5)	
T stage			0.927
1–2	141(24.1)	16(24.6)	
3–4	444(75.9)	49(75.4)	
N stage			0.063
0–1	323(55.2)	28(43.1)	
2–3	262(44.8)	37(56.9)	
Clinical stage			0.724
1–2	58(9.9)	5(7.7)	
3–4	527(90.1)	60(92.3)	
WHO type			0.350
2	29(5.0)	1(1.5)	
3	556(95.0)	64(98.5)	
EBV DNA			0.747
≥4000	357(61.0)	41(63.1)	
<4000	228(39.0)	24(36.9)	
Median follow-up months,median(range)	48.9(1.3–85.5)	50.2(2.5–84.0)	0.706

### Hemoglobin values

In the enrolled patients, the mean baseline Hb value of all participants was 14.1 g/dl, ranging from 7.5g/dl to 18.1 g/dl. Approximately 64 (9.8%) of patients had anemia before treatment. The mean Hb level during treatment was 12.9 g/dl, ranging from 7.4 g/dl to 15.9 g/dl. And the mean Hb level after treatment was 12.0 g/dl, with the range of 5.8 g/dl to 12.2 g/dl. There were 613 patients had Hb decreased after radiotherapy. The median Hb decrease was 2.2g/dl (range: 1.0–9.2 g/dl). We divided the Hb decreased patients into 2 groups: 295 (48.1%) had less than 2.2 g/dl, and 318 (51.9%) had ΔHb more than 2.2 g/dl (The cutoff value of ΔHb was based on the median of the maximum and minimum Hb value between pre-treatment and post-treatment). According to the Hb level before, during, and after treatment, 508 patients (78.2%) were divided into the Hb continuously decreased group (pre-treatment Hb> mid-treatment Hb> post-treatment Hb) and 142 patients (21.8%) into the non decreased group.

Age, the tumor size (T1-2, T3-4), the lymph node status (N0-1, N2-3), the clinical stage (1–2, 3–4), EBV DNA (<4000 copies/ml, ≥4000 copies/ml), the median follow-up time did not differ between the patients of no-anemia and anemia before radiotherapy, but there was a difference regarding gender (P<0.05) (Table 1).

### Treatment outcomes

Across all enrolled patients, 56 (8.6%) patients developed locoregional disease recurrence, 73 (11.2%) developed distant metastasis, and 26 (4.0%) died. The 5-year survival rates of all the 650 patients were as follows: OS, 88.2% and LRFS, 95.3%. The 5-year LRFS, and DMFS rates of the different groups including pre-treatment anemia vs. pre-treatment no-anemia, mid-treatment anemia vs. mid-treatment no-anemia, Hb continuous decrease vs. noncontinuous decrease, and ΔHb < 2.2g/dl vs. ΔHb ≥ 2.2 g/dl are shown in Table 2.

**Table 2:** The treatment outcome of the different groups. *Abbreviations*: Hb = hemoglobin; ΔHb = ΔHb = individual differences between pre-treatment hemoglobin and post-treatment; NA = not observed events; OS = overall survival; LRFS = local regional recurrence free survival; DMFS = distant metastasis-free survival.

	5-y OS (%)	*P*	5-y LRFS (%)	*P*	5-y DMFS (%)	*P*
Pre-treatment Hb levels		0.010		0.323		0.001
Anemia	80.7		98.2		76.2	
No anemia	89.1		95.0		88.9	
Mid-treatment Hb levels		0.004		0.141		0.052
Anemia	83.3		94.2		85.1	
No anemia	91.7		96.0		89.3	
Hb dynamic change		0.179		0.077		0.561
Continuous decrease	87.1		94.4		87.2	
Noncontinuous decrease	92.5		98.5		89.0	
ΔHb value		0.620		0.220		0.433
ΔHb<2.2g/dl	88.7		96.8		86.9	
ΔHb≥2.2g/dl	87.7		93.9		88.3	
Pre-treatment anemia		0.464		0.344		0.529
Continuous decrease	75.9		96.6		72.7	
Noncontinuous decrease	86.7		NA		80.1	
Pre-treatment no anemia		0.087		0.151		0.281
Continuous decrease	85.9		94.2		88.3	
Noncontinuous decrease	94.0		98.2		91.3	
Pre-treatment anemia		0.325		0.578		0.840
ΔHb<2.2g/dl	83.7		97.6		76.6	
ΔHb≥2.2g/dl	72.2		NA		75.0	
Pre-treatment no anemia			0.560	0.239		0.702
ΔHb<2.2g/dl	89.6		96.6		88.6	
ΔHb≥2.2g/dl	88.5		93.6		89.1	
Continuous decrease		0.844		0.535		0.447
ΔHb<2.2g/dl	86.9		95.7		86.4	
ΔHb≥2.2g/dl	88.2		95.8		87.8	
No-Continuous decrease		0.329		0.631		0.194
ΔHb<2.2g/dl	94.1		98.4		89.3	
ΔHb≥2.2g/dl	NA		NA		NA	

**Fig 1:**
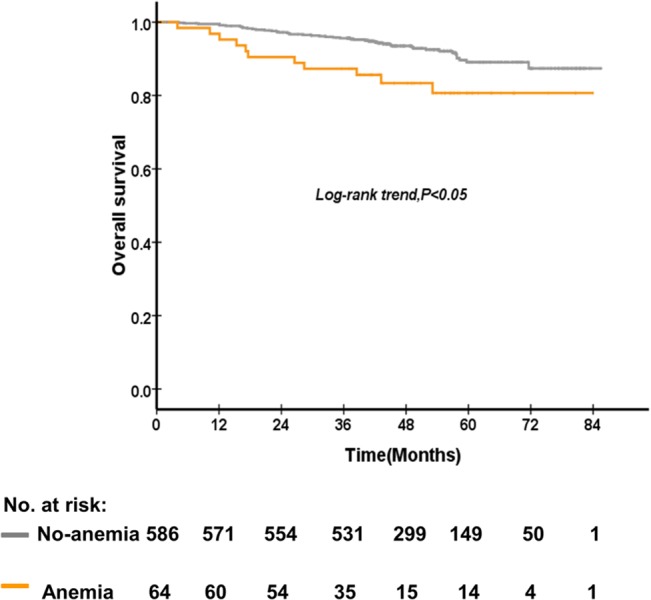
The comparison between patients of no-anemia and anemia before treatment on overall survival of 650 patients with nasopharyngeal carcinoma treated with concurrent chemoradiotherapy.

**Fig 2:**
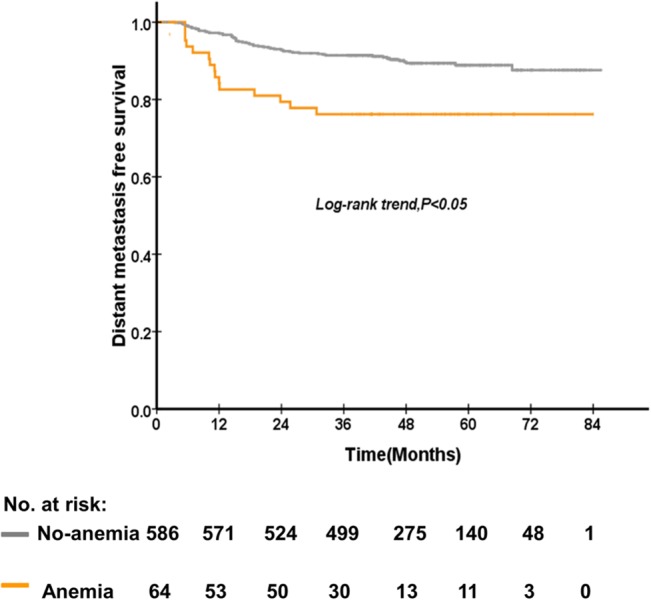
The comparison between patients of no-anemia and anemia before treatment on distant metastasis-free survival of 650 patients with nasopharyngeal carcinoma treated with concurrent chemoradiotherapy.

**Fig 3:**
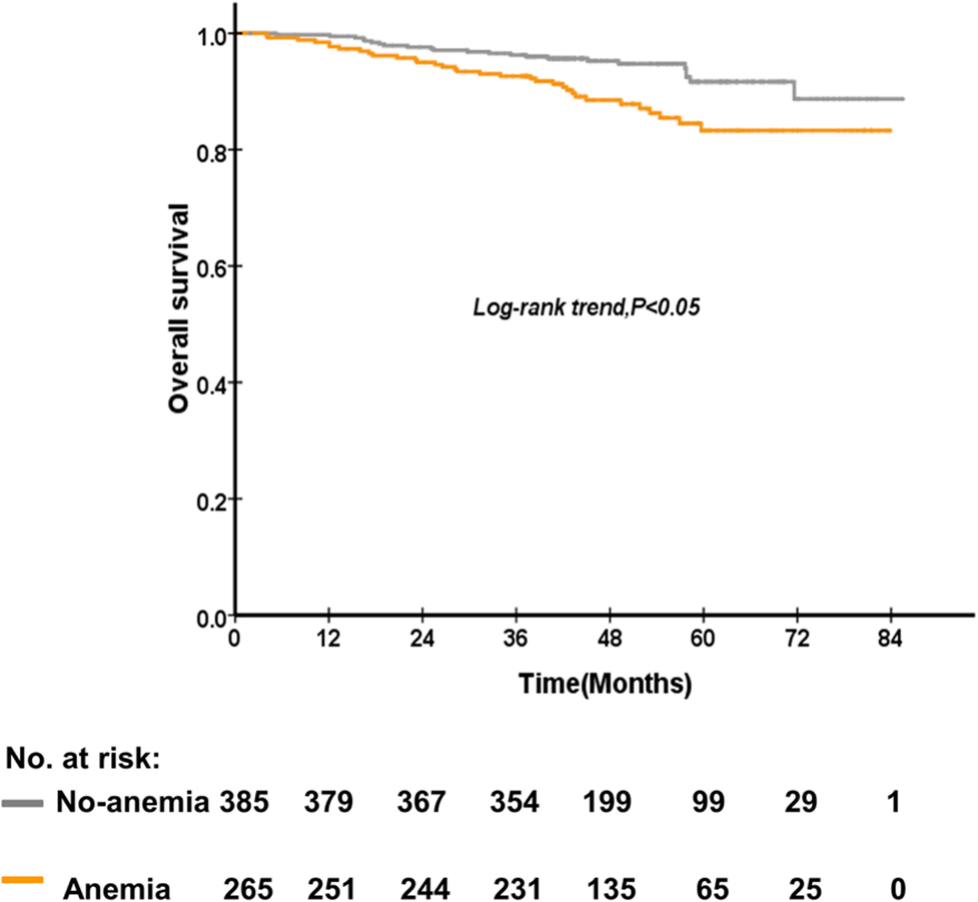
The comparison between patients of no-anemia and anemia during treatment on overall survival of 650 patients with nasopharyngeal carcinoma treated with concurrent chemoradiotherapy.

The 5-year OS rate of patients who were anemia and no-anemia before treatment were 89.1%, and 80.7% (P = 0.01), respectively (Fig 1). The 5-year DMFS rate of patients who were anemia and no-anemia before treatment were 88.9%, and 78.2% (P = 0.01) (Fig 2), respectively. On the other hand, the 5-year OS rate of patients who were anemia and no-anemia during treatment were 91.7% and 83.3% (P = 0.004) (Fig 3), respectively. However, post-treatment Hb level, Hb decrease level, and Hb continuous decrease were not associated with OS, LRFS and DMFS. No significant difference in OS, LRFS, and DMFS rate was found between anemia group and no-anemia group when patients were stratified according to pre-treatment and mid-treatment Hb levels, respectively. Besides, no significant difference in OS, LRFS, and DMFS rate was found between ΔHb<2.2g/dl and ΔHb≥ 2.2 g/dl group when patients were stratified according to pre-treatment and continuous decrease Hb levels, respectively.

Multivariate analysis was performed to adjust for various prognostic factors. Age (age ≤ 45 years, >45 years), gender, T stage, N stage, clinical stage, Epstein-Barr Virus (EBV) DNA (<4000 copies/ml, ≥4000 copies/ml), Hb continuous decrease (continuous decrease, no-continuous decrease), ΔHb (<2.2 g/dl, ≥2.2 g/dl), pre-treatment Hb level (anemia, no-anemia), mid-treatment Hb level (anemia, no-anemia), and post-treatment Hb level were regarded as factors in the multivariable analysis. According to multivariate analysis, the pre-treatment Hb level predicted a decreased DMFS (P = 0.007, HR = 2.555, 95% CI1.294–5.046) (Table 3). Besides, the mid-treatment Hb level predicted a decreased OS (P = 0.013, HR = 2.333, 95% CI1.199–4.541) (Table 4). Besides, gender and N stage were prognostic factors both for OS and DMFS. Pre-treatment Hb level (anemia or no-anemia), mid-treatment Hb level, post-treatment Hb level, Hb decrease and Hb continuous decrease were not associated with LRFS.

**Table 3:** Factors associated with OS by multivariate analysis in 650 patients. *Abbreviations*: Hb = hemoglobin; RR = relative risk; CI = confidence interval; ΔHb = individual differences between pre-treatment hemoglobin and post-treatment.

	*P*	RR	95.0% CI
Gender	0.002	3.534	1.560–8.005
Age	0.419	1.249	0.728–2.141
Pre-treatment Hb	0.079	1.975	0.923–4.225
Mid-treatment Hb	0.013	2.333	1.199–4.541
Post-treatment Hb	0.231	0.607	0.268–1.374
Hb continuous decrease	0.066	2.232	0.950–5.245
ΔHb	0.481	0.795	0.420–1.504
T stage	0.591	0.829	0.418–1.645
N stage	0.026	2.112	1.092–4.086
Clinical stage	0.954	0.958	0.227–4.038
EBV DNA	<0.001	5.008	2.589–9.689

**Table 4:** Factors associated with DMFS by multivariate analysis in 650 patients. *Abbreviations*: Hb = hemoglobin; RR = relative risk; CI = confidence interval; ΔHb = individual differences between pre-treatment hemoglobin and post-treatment.

	*P*	*RR*	*95*.*0% CI*
Gender	0.042	1.854	1.023–3.359
Age	0.860	0.959	0.603–1.526
Pre-treatment Hb	0.007	2.555	1.294–5.046
Mid-treatment Hb	0.218	1.438	0.806–2.564
Post-treatment Hb	0.341	0.727	0.376–1.403
Hb continuous decrease	0.117	1.709	0.874–3.341
ΔHb	0.237	0.715	0.410–1.247
T stage	0.610	0.846	0.445–1.608
N stage	0.266	1.370	0.786–2.389
Clinical stage	0.998	1.001	0.331–3.028
EBV DNA	<0.001	3.135	1.898–5.179

## Discussion

In the past years of clinical practice, the technology of IMRT resulted in satisfactory benefits for NPC patients in outcomes. Therefore, it is reasonable to reevaluate the predictive value of the Hb level for NPC in the new era of IMRT. This study revealed that pre-treatment anemia was an independent prognostic factor for a poor DMFS, and the mid-treatment anemia was a poor prognosticator for OS.

At the time of the current analysis, to our knowledge, no study had emphasized the prognostic value of the hemoglobin level in NPC patients treated with IMRT. The present study is the first study to evaluate the prognostic value of the hemoglobin level in NPC patients treated with IMRT. Our study analyzed 650 patients treated with IMRT, which is the largest population-based sample size.

This study revealed that anemia before treatment predicts poorer DMFS, with a 2.555-fold greater risk of disease metastasis. Yet the number of studies announced a relationship between anemia and disease metastasis was scarce. However, the exact mechanism remains uncertain. The decreased ability of the blood to carry oxygen due to low haemoglobin levels of less than 12 g/dl leads to tumor hypoxia [18]. The association between anemia and poor tumor oxygenation has been described in head and neck cancers [19]. Poor oxygenation occurs in most solid tumors and is correlated with a poor prognosis because of resistance to chemotherapy or radiotherapy caused by hypoxia and hypoxia-induced alterations to the molecular biology of the tumor. The HIF signalling pathway and the unfolded protein response activated by hypoxia have been reported to contribute to distant tumor metastasis [20].

We found that anemia during treatment led to a decreased OS and 2.333-fold higher risk in a multivariate analysis. Notably, given that few studies in the field of NPC have evaluated hemoglobin levels during radiotherapy and the important association between hemoglobin level and clinical outcome, more attention would be paid to hemoglobin level during radiotherapy among NPC patients. This result indicated that mid-treatment Hb changes may help improve the decision making process of anemia correction treatment in clinical settings. Control of the hemoglobin level during IMRT is expected to be a vital part of management among the therapeutic process in the clinical practices on NPC patients. However, since oral iron therapy always need a few weeks to reverse anemia, the amount of time elapsed between a low measured hemoglobin level and the attainment of a normal level of hemoglobin is currently too long [21]. The treatment of anemia induced by chemotherapy and radiotherapy includes red blood cell transfusions and erythropoiesis-stimulating agents [22, 23]. Consequently, hemoglobin corrections may contribute to better clinical outcomes, and future studies are expected to further confirm the role of hemoglobin corrections during treatments, such as transfusion and erythropoietin injection [24].

However, we did not find any association between Hb level and LRFS. The decrease in Hb level is not a prognostic factor for NPC patients treating with IMRT. Several researches revealed that anemia before treatment and during radiotherapy were prognostic factors for overall survival and local control on NPC patients at the time of conventional radiotherapy [11–13]. As we all know, local disease control in NPC patients is improved with the use of IMRT technology [14–16]. We surmise that Hb level did not have sufficient power to effect LRFS.

The mechanism by which anemia has influence on treatment outcomes is unknown. Hemoglobin level affects the oxygenation status in solid tumors, and an imbalance between the supply and consumption of oxygen can lead to tumor hypoxia [25]. Hypoxic areas are unevenly distributed in solid tumors with locally advanced stage [25]. A previous study showed that tumor hypoxia is common in patients with NPC and that tumor hypoxia has a role in tumor progression and resistance to treatment [26]. Solid tumors are resistant to radiation and some types of chemotherapyowing to tumor hypoxia [27, 28]. Furthermore,Alterations to gene expression and changes in the proteome and genome are induced by hypoxia. These changes may help tumor cells to survive in harmful microenvironments and nutrient deficiencies, thereby promoting cell proliferation in tumors. In addition, hypoxia is considered to induce resistance to X-rays, gamma-radiation and some types of chemotherapy, leading to treatment failures [25]. As shown in this study, hypoxia-induced decreases in the efficacy of chemotherapy and radiotherapy, together with anemia-induced nutritive deprivation, results in poorer overall survival as well as distant metastasis-free survival.

There are some limitations in this study. One is that it involved a single institution and that all the participants were enrolled from a single cancer center in an area with a high incidence of the disease. It is unclear whether the results of this study could be extrapolated to an area that the incidence of NPC is lower. Multicenter trials may be able to verify the value of the hemoglobin level in locoregional advanced nasopharyngeal carcinoma patients. Besides, the present study was a retrospective study. Further prospective research is needed to explore the value of Hb level in NPC treated with IMRT.

## Conclusions

Hemoglobin level is a useful prognostic factor in NPC patients receiving IMRT. We report that the pre-treatment Hb level is an independent prognostic factorfor DMFS. Besides, the mid-treatment Hb level is an independent prognostic factor for OS in NPC patients in the era of IMRT. However, the continuous decrease is not a prognostic factor for NPC patients treating with IMRT.
